# Electrophysiological correlates of the brain-derived neurotrophic factor (*BDNF*) Val66Met polymorphism

**DOI:** 10.1038/s41598-020-74780-9

**Published:** 2020-10-21

**Authors:** Nikita Roy, Robert J. Barry, Francesca E. Fernandez, Chai K. Lim, Mahmoud A. Al-Dabbas, Diana Karamacoska, Samantha J. Broyd, Nadia Solowij, Christine L. Chiu, Genevieve Z. Steiner

**Affiliations:** 1 NICM Health Research Institute and Translational Health Research Institute (THRI), Western Sydney University, Locked Bag 1797, Penrith, NSW 2751 Australia; 2 Brain & Behaviour Research Institute and School of Psychology, University of Wollongong, Wollongong, NSW 2522 Australia; 3 School of Health and Behavioural Science, Faculty of Health Sciences, Australian Catholic University, Brisbane, NSW 4014 Australia; 4 Faculty of Science, Medicine and Health, University of Wollongong, Wollongong, NSW 2522 Australia; 5 School of Psychology and Illawarra Health and Medical Research Institute, University of Wollongong, Wollongong, NSW 2522 Australia; 6 Faculty of Medicine, Health and Human Sciences, Macquarie University, Macquarie Park, NSW 2190 Australia

**Keywords:** Genetic association study, Genotype, Cognitive neuroscience, Genetics of the nervous system, Neurotrophic factors, Neurophysiology

## Abstract

The brain-derived neurotrophic factor (BDNF) protein is essential for neuronal development. Val66Met (rs6265) is a functional polymorphism at codon 66 of the *BDNF* gene that affects neuroplasticity and has been associated with cognition, brain structure and function. The aim of this study was to clarify the relationship between *BDNF* Val66Met polymorphism and neuronal oscillatory activity, using the electroencephalogram (EEG), in a normative cohort. Neurotypical (*N* = 92) young adults were genotyped for the *BDNF* Val66Met polymorphism and had eyes open resting-state EEG recorded for four minutes. Focal increases in right fronto-parietal delta, and decreases in alpha-1 and right hemispheric alpha-2 amplitudes were observed for the Met/Met genotype group compared to Val/Val and Val/Met groups. Stronger frontal topographies were demonstrated for beta-1 and beta-2 in the Val/Met group versus the Val/Val group. Findings highlight *BDNF* Val66Met genotypic differences in EEG spectral amplitudes, with increased cortical excitability implications for Met allele carriers.

## Introduction

Brain-derived neurotrophic factor (BDNF) is an essential protein widely expressed in the brain^1^ that is critical for neurogenesis and differentiation, synaptic plasticity, and cognition (learning, memory, executive functioning)^2^. The *BDNF* Val66Met (rs6265) polymorphism is a functional polymorphism in the BDNF gene where a valine (Val) to methionine (Met) amino acid substitution at codon 66^3^ occurs. The *BDNF* Val66Met polymorphism causes impaired activity-dependent secretion of BDNF in individuals carrying one or more copies of the Met allele relative to non-Met carriers (Val/Val genotype)^4^. The Met allele has been associated with cognitive impairment, particularly memory^5^, reduced hippocampal volume^6^, increased risk of depression^7,8^, anxiety-related behaviors^9^, neurodegenerative disorders^10^, particularly Alzheimer’s disease (AD), and psychiatric disorders such as schizophrenia^11^. Due to its neuroprotective effects, BDNF has also been implicated as a potential therapeutic agent for AD^12^.

The *BDNF* Val66Met polymorphism has been linked to memory function in healthy adults, with young adult Met carriers (Val/Met, Met/Met) exhibiting poorer episodic memory performance (as measured by the Repeatable Battery for the Assessment of Neuropsychological Status [RBANS]) than their Val homozygous peers^12^. Young adults (aged 24.0 ± 0.8 years) heterozygous for the Val/Met genotype also showed lower working memory ability (as measured by a 2-back task) when compared to the Val/Val homozygotes^4^.

There is also an established relationship between the *BDNF* Val66Met polymorphism, white and grey matter structural alterations, and functional hemodynamic brain changes in healthy individuals^3,13^. Structural magnetic resonance imaging (MRI) studies have reported a reduction in the volume of temporal and occipital lobe grey matter^14^, hippocampus^15^, amygdala^16^, and prefrontal cortex^15^ in healthy Met carriers (Val/Met, Met/Met) compared to Val/Val homozygotes of the *BDNF* Val66Met polymorphism. Healthy middle-aged adults (46.4 ± 6.8 years) carrying one or more copies of the Met allele showed lower hemodynamic responses (blood oxygen level dependent [BOLD]) in the right superior frontal gyrus and the middle occipital gyrus than the non-Met carriers (Val/Val genotype, aged 49.1 ± 4.1 years) during n-back working memory tasks, as measured by functional MRI (fMRI)^17^. Alterations in hippocampal and lateral prefrontal activation in Met/Met homozygotes, and a localised reduction in hippocampal grey matter volume compared to Val carriers (Val/Met and Val/Val genotypes), as measured by fMRI and structural MRI (respectively) have also been demonstrated^18^.

Resting state electroencephalographic (EEG) spectral activity provides a measurement of the intrinsic activity of neuronal populations and large-scale neuronal networks, and is linked to a range of factors such as cognitive function, task performance, clinical phenotypes, and arousal states. Several studies have explored the effects of the *BDNF* Val66Met polymorphism on waking resting state EEG activity in adults across several conditions. In healthy adults (aged 24.0 ± 0.8 years), EEG alpha power (9.75–11.75 Hz) in prolonged (40 h) wakefulness was roughly doubled in Val/Val homozygotes compared to the heterozygous counterpart (Val/Met genotypes)^4^. In another study of participants assessed for trait depression, homozygous Met allele carriers (aged 36.9 ± 12.6 years) exhibited higher delta (1.5–3.5 Hz) and theta (4.0–7.5 Hz) power, and lower alpha (8–13 Hz) and beta (14.5–30 Hz) power in both eyes-open (EO) and eyes-closed (EC) resting-state EEG, relative to participants homozygous for the Val allele^7^; this Met/Met-related modulation of neuronal activity was associated with trait depression. Similarly, another study found a negative correlation between parietal-occipital alpha power (8–13 Hz) and depression severity, which was maximal in the Met/Met genotype group^8^. To date, all prior studies investigating the effects of the *BDNF* Val66Met polymorphism on EEG frequency spectra have done so in a clinical context (e.g., sleep deprivation, depression), with no studies characterising *BDNF* Val66Met genotypic differences in EEG spectra in a neurotypical context. It should be noted that a range of other studies have investigated *BDNF* Val66Met polymorphism-related differences in EEG-derived event-related potentials (ERPs)^18–20^, EEG recorded during sleep^4^, or under general anesthetic^21^, magnetoencephalography (MEG)^22^ and non-invasive brain stimulation such as transcranial magnetic stimulation (TMS) and transcranial direct current stimulation (tDCS)^20,23–25^; these are beyond the scope of the current study and will not be reviewed further.

The aim of this study was to further understand the relationship between variants of the *BDNF* Val66Met polymorphism and EEG brain activity in neurotypical young adults. It was hypothesised that compared to the Val/Val genotype group, individuals carrying one or more copies of the Met allele (Met/Met and Val/Met genotype groups) would have greater delta and theta, and lower alpha activity^4,7^, and that the Val/Met genotype group would have greater beta activity than the Met/Met genotype group^7^. To improve comparability with previous EEG studies and test key hypotheses^7,8,18,19^, the three genotype groups were compared, rather than testing Met allele carriers versus non-Met carriers.

## Materials and methods

### Eligibility criteria

Individuals enrolled in the School of Psychology at the University of Wollongong were recruited to participate in the study in return for course credit. Written informed consent was obtained, followed by completion of a demographic and screening questionnaire. Saliva samples were collected using the OrageneDNA collection kit (DNA Genotek, Canada), and EEG measures were recorded. Participants were instructed to refrain from consuming psychoactive substances for at least 12 h prior to EEG testing, and from consuming tea, coffee, alcohol, and cigarettes for at least 2 h prior to saliva collection. Eligibility criteria were self-reported, and included no prior use of psychotropic/central nervous system medication, no neurological or psychiatric illnesses, normal hearing, and normal or corrected-to-normal vision. Ethical approval for this study was obtained from the University of Wollongong and Illawarra and Shoalhaven Local Health District Health and Medical Human Research Ethics Committee. This research was conducted in accordance with the International Ethical Guidelines for Biomedical Research Involving Human Subjects prepared by the Council for International Organisations of Medical Sciences (CIOMS) in collaboration with the World Health Organisation (WHO), and the Australian National Statement on Ethical Conduct in Research Involving Humans. Written informed consent was obtained from all study participants, and participants were free to withdraw at any time without penalty.

### DNA extraction and Val66Met genotyping

As per Steiner et al.^20^, following sample collection, saliva was stored at room temperature until DNA was extracted as per the manufacturer’s recommendations (Oragene, Canada). After extraction, DNA samples were quantified using a NanoDrop ND-1000, and stored at − 20 °C. High-throughput SNP genotyping was performed using the MassARRAY genotyping assay (Sequenom, Inc., San Diego, CA), with the analysis performed by matrix-assisted laser desorption/ionization time-of-flight mass spectrometry (MALDI-TOF MS). Extension primer design, and selection were performed using MassARRAY Designer Software (Sequenom, Inc., San Diego, CA). Control samples were run in parallel with the tested samples (on each plate), allowing for accurate evaluation and quality control checks of each of the steps involved (PCR amplification, Shrimp Alkaline Phosphatase treatment, extension PCR, nano-dropping to check product concentrations, nano-dispensing of samples onto the chip, and instrument performance). Each participant was genotyped as homozygous Val/Val (GG), heterozygous Val/Met (GA), or homozygous Met/Met (AA)^20^.

### EEG acquisition and processing

As per Steiner et al., 2018, participants were seated in an air-conditioned room 600–800 mm in front of a 19″ Dell LCD monitor, and completed an electrooculogram (EOG)/EEG calibration task^21^. Participants were then instructed to fixate on a 10 × 10 mm gray cross centered on a black background, while EO resting EEG activity was recorded for 4 min.

Continuous EEG data were recorded DC–30 Hz with a Neuroscan Synamps 2 digital signal-processing system and Neuroscan 4.3.1 Acquire software (Compumedics, Charlotte, NC) from 30 scalp sites (Fp1, Fp2, F7, F3, Fz, F4, F8, FT7, FC3, FCz, FC4, FT8, T7, C3, Cz, C4, T8, TP7, CP3, CPz, CP4, TP8, P7, P3, Pz, P4, P8, O1, Oz, O2), grounded by an electrode located midway between Fp1, Fp2, and Fz. Two recording setups were combined to obtain data: one used electrode caps with tin electrodes referenced to A1 and included A2 as a separate active channel for offline re-referencing (N = 59), sampled at 1000 Hz; the second setup was recorded using sintered Ag/AgCl electrodes referenced to the nose and recording M1 and M2 as active channels (*N* = 33) sampled at 2000 Hz. In both setups, EOG was recorded using tin cup electrodes placed 2 cm above and below the left eye for vertical movements, and on the outer canthus of each eye for horizontal movements. Impedance was < 10 kΩ for all electrodes.

EEG data were corrected for all eye-movement types using the Revised Aligned-Artifact Average (RAAA) EOG Correction Program^21^, which removes the EOG voltage contribution from the EEG using a regression-based approach (with coefficients based on the calibration task run for each participant). Data were re-referenced to digitally linked ears (*N* = 59) or mastoids (*N* = 33) and extracted offline using Neuroscan Edit software (Compumedics, Charlotte, NC); the dataset recorded at 2000 Hz was resampled to 1000 Hz. The 4-min of data were segmented into 2000 ms epochs, each DC-corrected by baselining across the entire epoch, and any epochs containing artifact exceeding ± 75 μV were rejected. A visual inspection was conducted to further remove artifact-contaminated trials, and interpolate channels containing excessive artifact (> 70% epochs retained) with unaffected data from immediately surrounding sites. Data were quantified in MATLAB and EEGLAB (Version 14.0.0b). For each subject and epoch, spectral amplitudes were computed using Discrete Fourier Transforms with a 10% Hanning window; correction for the window was subsequently applied. Frequency data from 0.0 to 29.5 Hz were extracted with a resolution of 0.5 Hz before averaging across epochs. The spectral band amplitudes were calculated by summing the activity across the frequency bins for delta: 0.5–3.5 Hz; theta: 4.0–7.5 Hz; alpha-1: 8.0–10.5 Hz; alpha-2: 11.0–13.5 Hz; beta-1: 14.0–20.5 Hz; and beta-2: 21.0–29.0 Hz. This resulted in one datapoint for each subject, electrode, and band.

### Statistical analyses

**Figure 1:**
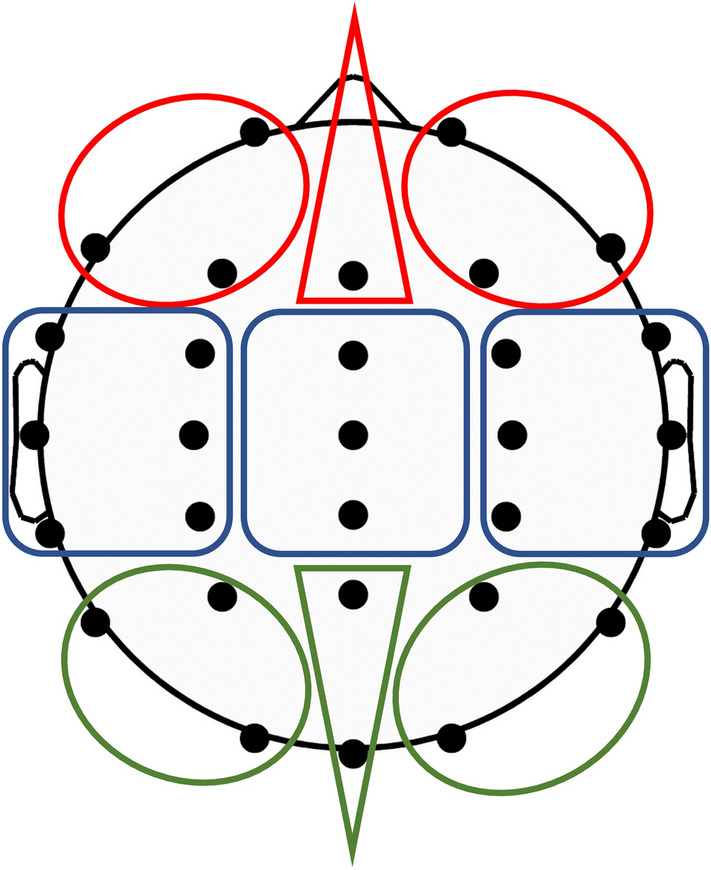
Regions of interest to facilitate topographic analyses of EEG bands.

Mixed-model 3 × 3 × 3 MANOVAs with the within-subjects factors of sagittal (frontal, central, parietal) and coronal topographies (left, midline, right) and between-subjects factor of genotype (Val/Val, Val/Met, Met/Met) were conducted for each EEG band using IBM SPSS Statistics, v22. To facilitate topographic analyses of EEG bands, data were pooled across the 30 electrodes to form 9 regions: frontal-left (FL: Fp1, F3, FC3,F7, FT7), frontal-midline (FM: Fz, FCz), frontal-right (FR: Fp2, F4, FC4, F8, FT8); central-left (CL: C3, CP3, T7, TP7), central-midline (CM: Cz, CPz), central-right (CR: C4, CP4, T8, TP8); posterior-left (PL: P3, P7, O1), posterior-midline (PM: Pz, Oz), posterior-right (PR: P4, P8, O2). These regions are represented diagrammatically in Fig. 1. EEG band topographies from these 9 regions (FL, FM, FR, CL, CM, CR, PL, PM, PR) were compared using planned orthogonal contrasts within each plane – sagittal: frontal (F) versus posterior (P) regions, and central (C) versus the mean of frontal and posterior regions (F/P); and coronal: left (L) versus right (R), and midline (M) versus the mean of left and right regions (L/R). Genotypes were compared using planned simple contrasts: Val/Val *versus* Met/Met, Val/Val *versus* Val/Met, and Val/Met *versus* Met/Met. All *F*-tests reported were one-tailed (because directional hypotheses were specified) and had (1, 89) degrees of freedom unless otherwise specified; α was set at 0.05 for all tests. It should also be noted that as this paper details results for multiple dependent measures (e.g., EEG frequency bands), the frequency of Type I errors increases. However, this increase in frequency of Type I errors cannot be controlled by adjusting α-levels, because the probability of Type I error remains the same^26^.

eLORETA (exact low resolution brain electromagnetic tomography, v20170220^27,28^ was used to examine the sources of each EEG frequency band. Source localisation was conducted on the grand mean EEG band amplitudes with the aim of complementing the statistical analyses of their scalp topography and assist with the neurobiological interpretation of the results.

### Ethics approval

Ethics approval for this study was requested and approved through the University of Wollongong and Illawarra and Shoalhaven Local Health District Health and Medical Human Research Ethics Committee. This research was conducted in accordance with the International Ethical Guidelines for Biomedical Research Involving Human Subjects prepared by the Council for International Organisations of Medical Sciences (CIOMS) in collaboration with the World Health Organisation (WHO), and the Australian National Statement on Ethical Conduct in Research Involving Humans.

### Consent to participate

All participants were provided with a copy of the participant information sheet and consent form, and written informed consent was obtained from all participants prior to ocmmencing data collection.

### Consent for publication

All participants consented to the publication of their data in the deidentified aggregate form in which it is presented.

## Results

### Genotype

The sample included 92 undergraduate students (*M*_age_ = 21.4, *SD* = 5.0 years, 67% female, majority Caucasian ethnicity). Table 1 shows that there were no significant differences in age, gender, lab setup (as the data were recorded in two different labs; see Sect. 2.3), or number of epochs accepted between the three *BDNF* Val66Met genotype groups (all *p* > 0.05). Data were normally distributed based on the skewness and kurtosis of histogram plots. Genotype breakdowns across the sample were as follows: Val/Val *n* = 61 (66.3%); Val/Met *n* = 25 (27.2%); Met/Met *n* = 6 (6.5%). The allele frequency for G (encodes Val) was 74.6%, and A (encodes Met) was 25.4%. This did not differ from the Hardy–Weinberg equilibrium (*χ*^2^ = 0.64, *p* = 0.726).

**Table 1:** Age, gender, lab set-up, and epochs accepted information for *BDNF* Val66Met genotype groups.

	Val/Val (*n* = 61)	Val/Met (*n* = 25)	Met/Met (*n* = 6)	Statistical difference
Age, years	21.37 ± 5.11	21.69 ± 5.13	20.69 ± 3.19	*F*(2,91) = 0.10, *p* = 0.901
Gender Female *n* (% of total)	41 (67.21)	17 (68.00)	4 (66.67)	*χ*^2^(2) = 0.01, *p* = 0.996
Lab Setups	A = 20, B = 41	A = 10, B = 15	A = 3, B = 3	*χ*^2^(2) = 1.01, *p* = 0.603
Epochs accepted *n*	85.62 ± 21.44	84.48 ± 23.04	84.83 ± 26.06	*F*(2,91) = 0.03, *p* = 0.970

### EEG

**Figure 2:**
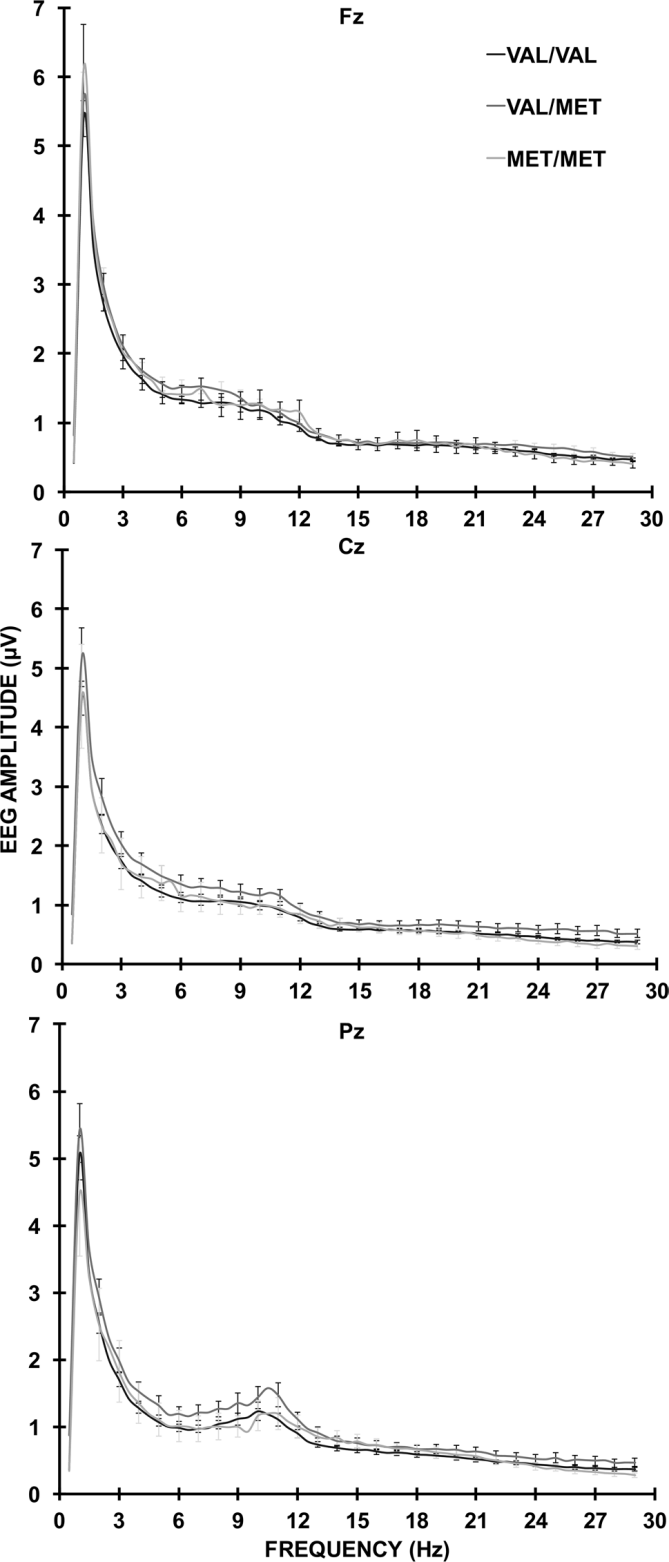
EEG Spectra at Fz, Cz, and Pz for each of the *BDNF* Val66Met genotypes (Val/Val, Val/Met, Met/Met).

EEG spectral data for the two lab setups across the 30 scalp sites and six EEG bands were highly similar (*r*(180) = 0.80, *p* < 0.001); thus data were pooled and not treated separately for any further analyses. EEG spectra for each of the three *BDNF* Val66Met genotype groups at Fz, Cz, and Pz are shown in Fig. 2. The MANOVA outcomes that were statistically significant for each of the EEG bands are summarised below in text with the statistical outcomes in Table 2. Note that some of the pairs of greater than (>) and/or less than (<) sign entries in Table 2 are reversed in their text descriptions. This utilises the logical equivalence of such pair reversals, facilitating the tabulation of band differences in each effect (e.g., C < F/P × M > L/R is logically equivalent to C > F/P × M < L/R).

**Table 2:** Statistically significant (*p* < .05) outcomes from the mixed-model MANOVAs.

Band	Effect	*F*	*p*	η_p_^2^
Delta	F > P	9.962	.002	.10
	L < R	6.892	.010	.07
	M > L/R	25.209	< .001	.22
	F > P × M > L/R	23.294	< .001	.21
	C < F/P × L < R	19.299	< .001	.18
	Val/Val < Met/Met × L < R	7.101	.009	.07
	Val/Met < Met/Met × L < R	8.732	.004	.09
	Val/Val < Met/Met × C < F/P × L < R	6.098	.015	.06
	Val/Met < Met/Met × C < F/P × L < R	8.223	.005	.08
Theta	F > P	30.581	< .001	.25
	M > L/R	46.922	< .001	.34
	F > P × M > L/R	139.727	< .001	.61
	C < F/P × L < R	7.858	.006	.08
Alpha-1	F < P	13.739	< .001	.13
	L < R	4.936	.029	.05
	M > L/R	18.515	.000	.17
	F > P × M > L/R	43.829	< .001	.32
	C < F/P × L < R	11.423	.001	.11
	Val/Val < Met/Met × C < F/P × L < R	5.034	.027	.05
	Val/Met < Met/Met × C < F/P × L < R	4.805	.031	.05
Alpha-2	F < P	24.126	< .001	.21
	L < R	7.137	.009	.07
	M > L/R	8.388	.005	.08
	F > P × M > L/R	39.276	< .001	.30
	C > F/P × L > R	5.141	.026	.05
	C > F/P × M > L/R	15.351	< .001	.14
	Val/Val < Met/Met × L < R	6.478	.013	.08
	Val/Met < Met/Met × L < R	6.312	.014	.07
Beta-1	F < P	14.236	< .001	.13
	C < F/P	5.675	.019	.05
	F > P × M > L/R	41.346	< .001	.31
	C < F/P × M > L/R	4.166	.044	.04
	Val/Val < Val/Met × F > P	7.416	.008	.08
Beta-2	F > P	11.097	.001	.11
	C < F/P	14.704	< .001	.14
	F > P × M > L/R	5.446	.022	.05
	Val/Val < Val/Met × F > P	4.448	.038	.05

**Figure 3:**
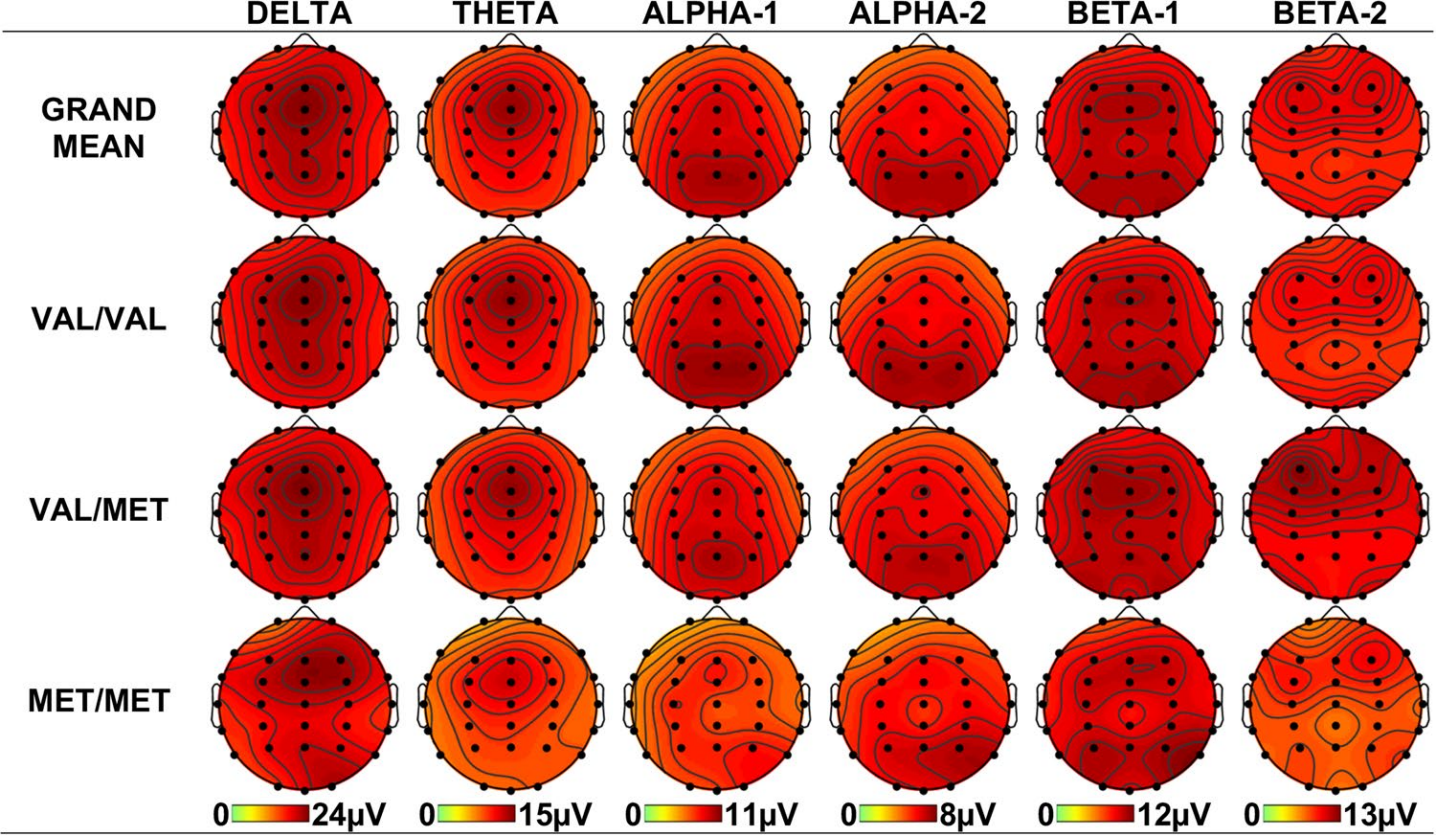
Topographic headmaps for each of the six spectral bands. The top row shows the grand mean across the three genotypes, and the lower three rows demonstrate the across-subjects means for each genotype separately (Val/Val, Val/Met, Met/Met). Parieto-right differences for delta and alpha-1, and right hemispheric differences for alpha-2 can be seen between the Met/Met genotype group and both the Val/Val and Val/Met genotype groups, and frontal beta-1/2 differences for Val/Met compared to Val/Val genotype groups.

Delta amplitudes were greater frontally than parietally (F > P: *F* = 9.962, *p* = 0.002, η_p_^2^ = 0.10), larger on the right than the left (L < R: *F* = 6.892, *p* = 0.010, η_p_^2^ = 0.07), and in the midline compared with the hemispheres (M > L/R: *F* = 25.209, *p* < 0.001, η_p_^2^ = 0.22). Frontal amplitudes were greatest in the midline (F > P × M > L/R: *F* = 23.294, *p* < 0.001, η_p_^2^ = 0.21), and fronto-posterior amplitudes were greatest on the right (C < F/P × L < R: *F* = 19.299, *p* < 0.001, η_p_^2^ = 0.18). As shown in Fig. 3 the right hemispheric enhancement was larger for the Met/Met genotype group compared with the Val/Val and Val/Met genotype groups (Val/Val < Met/Met × L < R: *F* = 7.101, *p* = 0.009, η_p_^2^ = 0.07; Val/Met < Met/Met × L < R: *F* = 8.732, *p* = 0.004, η_p_^2^ = 0.09), as was the right fronto-posterior enhancement (Val/Val < Met/Met × C < F/P × L < R: *F* = 6.098, *p* = 0.015, η_p_^2^ = 0.06; Val/Met < Met/Met × C < F/P × L < R: *F* = 8.223, *p* = 0.005, η_p_^2^ = 0.08). To improve readability, statistical effects are omitted from the remaining text; please refer to Table 2 for this information.

Theta amplitudes showed frontal (*p* < 0.001) as well as midline enhancements (*p* < 0.001); these effects interacted, with frontal amplitudes greatest in the midline (*p* < 0.001). Theta was greater on the right fronto-posteriorly (*p* = 0.006). There were no main effects or interactions involving *BDNF* Val66Met genotype.

Alpha-1 amplitudes were largest in the posterior region (*p* < 0.001), in the right hemisphere (*p* = 0.029) and in the midline (*p* < 0.001), with the midline enhancement largest frontally (*p* < 0.001). Alpha-1 was smaller centrally on the right (*p* = 0.001). This right-central reduction in alpha-1 was greater for Met/Met than for both Val/Val and Val/Met genotype groups (*p* = 0.027, *p* = 0.031).

Alpha-2 showed a parietal topography (*p* < 0.001) that was greatest on the right (*p* = 0.009). There was a midline enhancement (*p* = 0.005) that was largest frontally (*p* < 0.001) and centrally (*p* < 0.001); central alpha-2 was larger in the left than right hemisphere (*p* = 0.026). The Met/Met group showed a reduction in this left hemispheric enhancement observed for both Val/Val and Val/Met genotype groups (*p* = 0.013, *p* = 0.014).

For Beta-1, amplitudes were maximal in the parietal region (*p* < 0.001), and fronto-parietally compared to centrally (*p* = 0.019); this was greatest in the midline (*p* = 0.044). Frontally, the interaction was largest in the midline compared to the right and left hemispheres (*p* < 0.001). The Val/Met genotype group showed greater frontal beta-1 activity compared to the Val/Val genotype group (*p* = 0.008).

Beta-2 was enhanced frontally (*p* = 0.001) and fronto-parietally (*p* < 0.001), with the frontal enhancement being greatest in the midline (*p* = 0.022). As with beta-1, beta-2 amplitudes were larger frontally in the Val/Met genotype group compared to the Val/Val genotype group (*p* = 0.038).

### Source localisation

**Figure 4:**
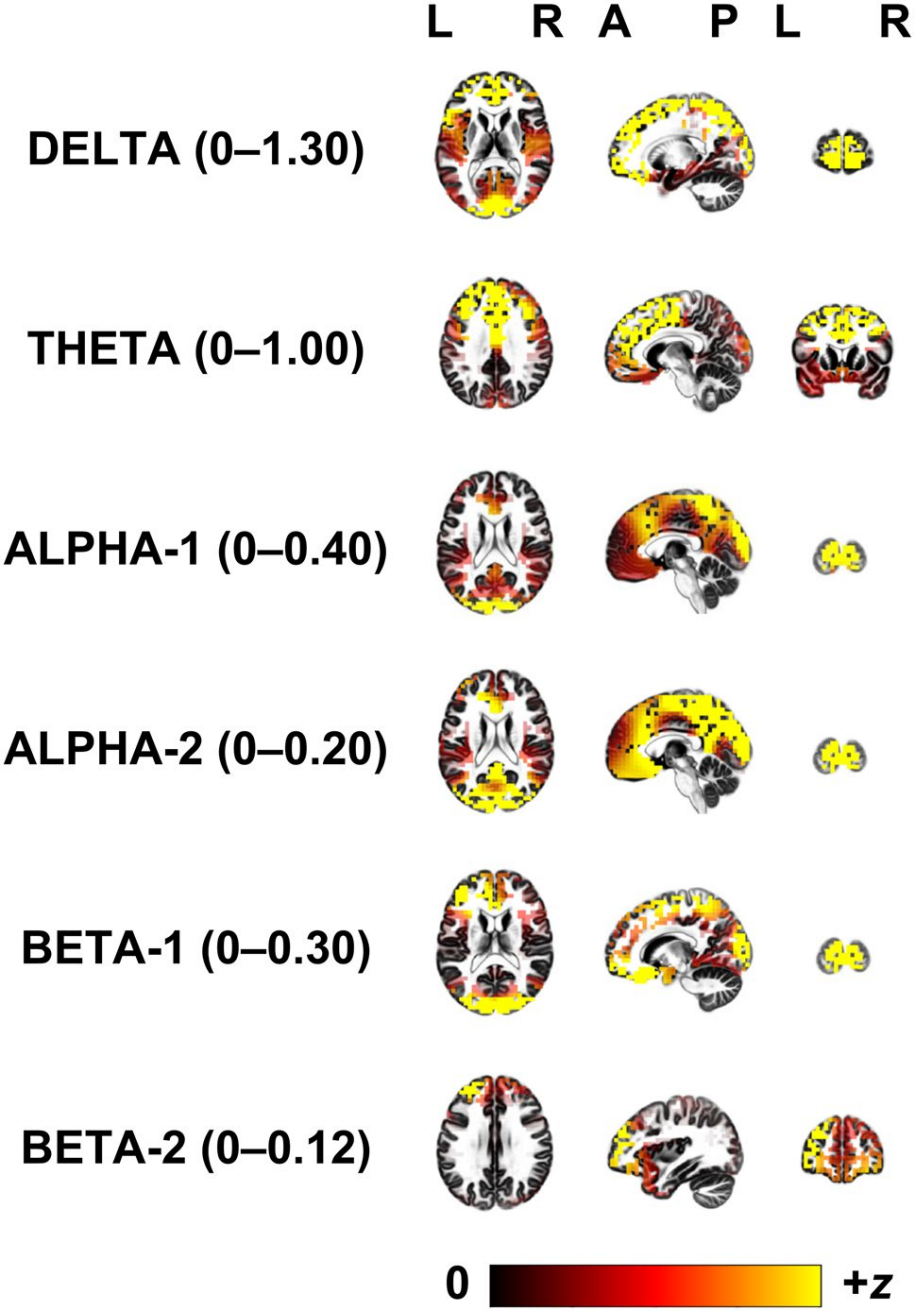
eLORETA source localisations for the grand mean amplitudes for each EEG band for horizontal, sagittal, and coronal planes. Source amplitudes are plotted as z-scores, and the scale for each band is to the right of the band name. L = Left; R = Right; A = Anterior; P = Posterior.

The grand mean eLORETA source plots for each of the EEG bands are illustrated in Fig. 4. The major structural sources and the dominant five Brodmann areas (BAs) involved for each band are detailed below. For delta, this was the superior and middle frontal gyrus (BA 9, 10, 11), inferior frontal gyrus (BA 47), and the anterior cingulate (BA 32). Theta was maximal in the cingulate gyrus (BA 6, 24, 32) and medial frontal gyrus (BA 8, 9). Alpha-1 was largest in the cuneus (BA 17, 18, 19), the paracentral lobule (BA 4), and the postcentral gyrus (BA 5). Alpha-2 was also maximal in the cuneus (BA 18, 19) the paracentral lobule (BA 4), and the postcentral gyrus (BA 3, 5). The five major sources active for beta-1 were the cuneus (BA 17, 18, 19), the superior frontal gyrus (BA 9), and the middle frontal gyrus (BA 10). The greatest sources for beta-2 were the superior frontal gyrus (BA 9, 10), the middle frontal gyrus (BA 11, 46), and the inferior frontal gyrus (BA 47).

## Discussion

This study explored the differences in resting state EEG spectral activity associated with the *BDNF* Val66Met polymorphism in healthy young adults. Hypotheses were partially confirmed, with the Met/Met genotype group showing enhanced fronto-parietal-right delta and attenuated right-centro-parietal alpha-1 and left hemispheric alpha-2 activity compared to both Val/Val and Val/Met genotype groups; unexpectedly, theta was unaffected by genotype status^4,7^. Counter to expectations, greater frontal beta-1 and beta-2 amplitudes were observed in the Val/Met genotype group compared to the Val/Val genotype group^7^. Findings have implications for increased cortical excitability for Met allele carriers that are discussed further below.

Right hemispheric and fronto-parieto-right delta amplitudes were greater for the Met/Met group compared to both homozygous Val/Val and heterozygous Val/Met genotypes. These genotype-related topographic differences seem to be driven by a right-parieto enhancement and a left hemispheric reduction for the Met/Met genotype group (see Fig. 3). This is in line with prior research that showed temporal and parieto-occipital differences in the Met/Met genotype group^7^. eLORETA identified a series of frontal sources for delta in the superior and middle frontal gyrus, inferior frontal gyrus, and the anterior cingulate. Interestingly, left anterior cingulate abnormalities (reduced glucose metabolism and volume) have been linked to schizotypal personality disorder and schizophrenia^29^. Further work is required to ascertain whether the left hemispheric delta reduction observed here in healthy young *BDNF* Met homozygotes is indeed linked to such left anterior cingulate abnormalities, and whether this confers increased psychosis risk^11^. Of note, the presence of the Met allele variant has also been shown to have functional consequences in individuals with chronic aphasia; it may play a role in speech and language, which is largely laterialised. In a sample of individuals with chronic aphasia as a result of left hemisphere stroke, a naming task revealed a greater number of activated voxels in the right hemisphere of Val/Val homozygotes than Met/Met individuals^30^. Future studies may seek to characterise whether healthy Met allele carriers demonstrate lateralised differences in speech and language processing mechanisms.

Theta amplitudes showed the typical midline frontal distribution, and this was reflected in the eLORETA source plots with diffuse sources in the cingulate gyrus and medial frontal gyrus. Counter to our hypothesis, there were no effects of genotype on theta amplitudes^4,7^. One previous study reported elevated theta power in the Met/Met genotype group as opposed to Val/Val homozygotes^7^, while another showed that theta is reduced in Met allele carriers during REM sleep and resting state EC conditions^4^. However, these prior studies were in the context of depression and sleep deprivation and elicited theta amplitudes during sleep states and a memory task, where theta has been shown to be maximal^31^. This suggests that a resting-state, eyes-open paradigm in healthy individuals may not be optimal to observe theta wave amplitude differences associated with BDNF rs6265.

Consistent with our hypothesis, the Met/Met genotype group exhibited reduced centro-parietal alpha-1 and left hemispheric alpha-2 amplitudes relative to both the Val/Val and Val/Met genotype groups. These results align with prior research which demonstrated that the Met/Met genotype group had reduced alpha power (8–13 Hz) compared to the Val/Val genotype group for temporal and parieto-occipital regions^7^. eLORETA analyses matched the dominant alpha-1/2 midline parietal distribution, with maximal sources in the cuneus, the paracentral lobule, and the postcentral gyrus. Alpha amplitudes largely originate in default mode network (DMN) functional hubs within the parietal and occipital lobes^32^ and have been linked with inhibition of active occipital cortical processing. The lower alpha-1 and alpha-2 amplitudes observed here in the Met/Met genotype group may point to dysregulation of occipital network processing, with less deactivation of active processing in DMN occipital hubs during an eyes open resting state^33^. Given that Met allele carriers of the *BDNF* Val66Met polymorphism have an increased risk of AD, it is important to note that reductions in alpha power (7.5–12.5 Hz) have also been linked to an earlier age of AD onset^34^, reflecting dysregulated cortical excitability most likely associated with excitotoxicity^35^. Future work is necessary to understand if reduced alpha power associated with the *BDNF* Val66Met polymorphism is also an early risk factor for cognitive impairment.

Our study was the first to observe greater frontal beta-1 and beta-2 amplitudes for the Val/Met genotype group than for the Val/Val genotype group. Prior research has revealed enhanced beta power in the Met/Met genotype group compared to the Val/Met genotype group (*n* = 13 per group), however our investigation did not replicate these findings^7^. This may suggest an additive effect of Met allele frequency on BDNF activity and beta, although this is speculative and requires replication in a study with balanced genotype groups. Beta activity reflects a state of cortical activation via excitatory pyramidal cells mediated by inhibitory interneurons gated by γ-aminobutyric acid type A (GABA_A_) receptors that act as ‘pacemakers’ to maintain beta and gamma rhythms^36–38^, suggesting that Met allele carriers may have a disequilibrium of excitatory/inhibitory homeostatic inputs in these frontal neural networks^39^. eLORETA analysis demonstrated maximal beta-1 sources in the superior and middle gyrus, and maximal beta-2 sources in the superior, middle, and inferior gyrus, suggesting that these may be key areas of interest for future studies investigating altered GABA_A_ receptor expression/activity in Met allele carriers, particularly in light of BDNF being shown to reduce GABA-ergic function via downregulation of GABA_A_ receptor expression^40^. This relationship may be further mediated through impairment of synaptic transmission and plasticity in *BDNF* Met/Met genotypes, as prior research has demonstrated a decrease in GABA_A_ receptor-mediated synaptic transmission in the pyramidal neurons of *BDNF* Met/Met mice^41^. Future work is required to understand if these findings are specific to particular brain regions and if there are functional effects of this altered receptor status. Greater beta amplitudes observed in the Met/Met genotype group may have functional implications in low-arousal states, and serve as a means of compensation for impaired task performance in a state of low arousal^42^. Although our study was conducted in a resting state, this finding could highlight a functional consequence of altered beta-1 and beta-2 activity in the Met-carrying groups.

### Strengths, limitations, conclusion

Factors that point to increased generalisability of our study include a robust sample size (*N* = 92), and no differences in age, sex, or data recording experimental set-ups. Given that our sample consisted of healthy neurotypical adults, rather than individuals that are part of a clinical group, normative data for resting-state EO EEG is presented by our study. Additionally, we conducted eLORETA source localisation for each EEG band, which adds depth and breadth to our EEG spectral findings. Nevertheless, our study had several limitations. We chose to sum across narrow frequency bands to form each of the “classic” EEG bands, with the additional subdivisions of alpha-1/2 and beta-1/2. Although these band limits aligned with previous work, subdivision selection nonetheless remained arbitrary. Future work should seek to use a more data-driven approach by utilising principal components analysis (PCA) to decompose the EEG frequency spectra^23,43^. Further, data-driven approaches such as cluster-based permutation can be applied to identify significant clusters across both the spatial and frequency domains^44^. We did not measure cognition in this cohort, so findings can only be interpreted in the context of intrinsic brain function. Although our study did not utilise a clinical sample, it does provide useful insights that may pinpoint early differences in brain function at a single timepoint amongst (currently) neurotypical individuals, that could help predict future changes in brain function and disease risk; this should be researched in future longitudinal studies. Finally, our study chose to isolate the homozygous Met/Met group from the heterozygous Val/Met group, rather than pooling the two together as ‘Met allele carriers’. Although this reduced study power due to the low sample size of the Met/Met genotype group (*n* = 6), it increased our study’s comparability to existing literature that chose to isolate Met/Met separately and had similar genotype group sizes to ours^7,8,18,19^. Obtaining a larger sample of participants who are homozygous for the Met allele in an effort to balance the sample size of each genotype group may further strengthen the power and significance of future studies. Such research would require a minimum genotype group size of 23 to detect a moderate to large effect size similar to prior studies^7,19^. From an epidemiological perspective, the population prevalence of Met/Met genotypes is relatively low: ~ 4.5% in prior studies, ~ 6.5% here, which would translate to a sample size of > 500.

This study examined the relationship between the *BDNF* Val66Met polymorphism and neuronal oscillatory activity. Findings demonstrate enhanced delta, reduced alpha-1 and alpha-2 in Met/Met homozygotes relative to Val allele carriers, enhanced beta-1 and beta-2 amplitudes in the Val/Met genotype group compared to the Val/Val genotype group, and an absence of any genotypic association with theta. Our findings confirm that varied *BDNF* genotypic presentation is important in understanding resting-state EO oscillatory EEG activity in healthy, neurotypical adults. Findings suggested dysregulated cortical excitability/inhibition in Met allele carriers; future work is required to further understand these physiological mechanisms and establish an association with cognition. This study highlights the importance of genetics as a factor that influences intrinsic brain activity, and confirms the utility of investigating genetics and neurophysiology in a neurotypical sample in future studies.

## Data Availability

The datasets generated and analysed during the current study are not publicly available because data could potentially result in the reidentification of participants due to small group sizes. Aggregate data can be made available upon reasonable request.

## Code availability

MATLAB scripts can be made available upon reasonable request.
